# 5-Aza-2′-deoxycytidin (Decitabine) increases cancer-testis antigen expression in head and neck squamous cell carcinoma and modifies immune checkpoint expression, especially in CD39-positive CD8 and CD4 T cells^☆^

**DOI:** 10.1016/j.neo.2024.101086

**Published:** 2024-11-27

**Authors:** Adrian Fehn, Adrian von Witzleben, Ayla Grages, Tsima Abou Kors, Jasmin Ezić, Annika C. Betzler, Cornelia Brunner, Patrick J. Schuler, Marie-Nicole Theodoraki, Thomas K. Hoffmann, Simon Laban

**Affiliations:** a Head and Neck Cancer Center of the Comprehensive Cancer Center, Department of Otorhinolaryngology and Head & Neck Surgery, Ulm University Medical Center, Germany; b Ulm University Medical Faculty, Core Facility Immune Monitoring, Ulm, Germany

**Keywords:** OPSCC, HPV, Immune-Checkpoint-Molecules, DNA-Methylation, Cancer Testis Antigens, Decitabine

## Abstract

Failure of immunotherapy in head and neck squamous cell carcinoma (HNSCC) patients represents an unmet need to augment leverage of adaptive immunity. Immunogenic cancer-testis antigen (CTA) expression as well as lymphocyte differentiation and function are regulated by DNA methylation. Therefore, epigenetic therapy via inhibition of DNA-Methyltransferases by 5-Aza-2′-deoxycytidine (DAC) serves a promising adjuvant in immunotherapy.

We investigated the effects of DAC on CTA expression and proliferative capacity in HNSCC cell lines and on the expression of 12 immune checkpoint molecules (ICM) on lymphocytes of oropharyngeal squamous cell carcinoma (OPSCC) patients and healthy donors.

In all cell lines CTA were upregulated accompanied by decreased proliferation. In lymphocytes pronounced alterations of the ICM repertoire were observed, influenced by donor type and subpopulation. On CD39+ CD4 and CD8 T cells, the expression of co-stimulatory ICM GITR and OX40 increased dose dependently, whereas expression decreased on CD39- CD4 T cells. PD1 expression increased primarily on CD39+ CD8 T cells and decreased on CD39- CD4 T cells. CD27 expression decreased primarily in CD8 T cells, but increased in CD39- CD4 T cells, whereas ICOS expression was lowered in both CD39+ and CD39- subsets of CD4 as well as CD8 T cells.

DAC treatment increased immunogenicity and decreased proliferation in HNSCC cells while enhancing expression of co-stimulatory ICM GITR and OX40. We propose low dose DAC treatment as a adjuvant to immunotherapy.

## Graphical abstract

**Figure:**
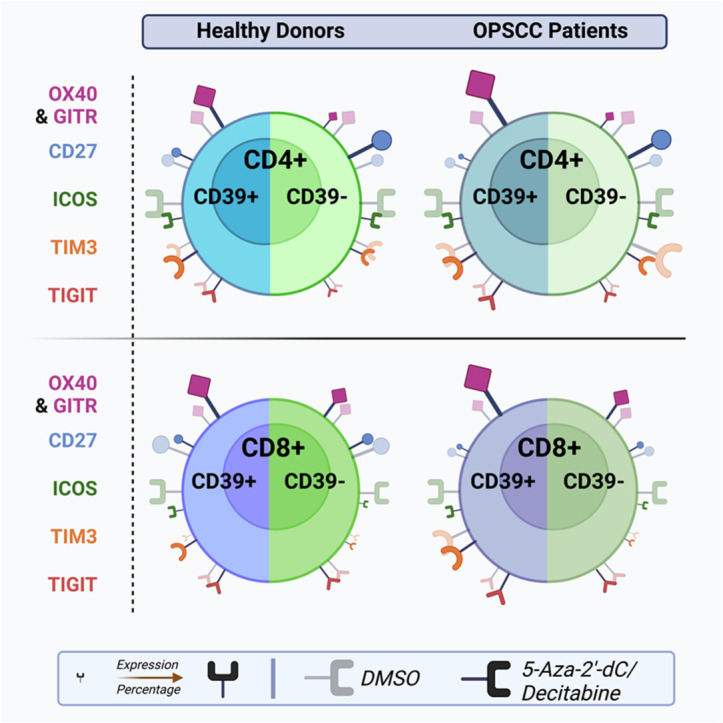

## Introduction

Immune checkpoint blockade (ICB), foremost PD-1/PD-L1 has been approved for recurrent / metastatic OPSCC [1], but only <20 % of patients responding [2] are indicative of a yet unmet need. Therefore, strategies to enhance immunogenicity of HNSCC are needed. DNA methylation is a fundamental regulatory epigenetic mechanism that plays a crucial role in lymphocyte differentiation.[3] Likewise, cancer cells exploit the epigenetic machinery to shape the tumor microenvironment (TME) and promote immune evasion. Epimutations need to be actively maintained and therefore serve as a promising target.[4] The epigenetic modifier 5-Aza-2′-deoxycytidin/Decitabine (DAC), has regained interest and was FDA approved for the treatment of myeloid malignancies.[5] DAC acts as suicide DNMT inhibitor.[6]

Demethylating therapy has been shown to restore the expression of tumor suppressor genes and de-repress cancer-testis antigens (CTA), endogenous retroviral elements, which can help to prevent or slow down the growth of cancer cells and increase immunogenicity.[7,8] In cancers, CTA have become an attractive target for cancer immunotherapy because they are selectively expressed in cancer cells and not in normal tissues. CTA are known to be expressed in HNSCC and often members of the MAGE family as well as the New York Esophageal squamous cell carcinoma 1 antigen (NY-ESO-1) are detected.[9] Yet, in HPV-negative HNSCC, the expression of MAGEA family antigens, and NY-ESO-1[9,10] as well as antibodies against MAGEA1 and MAGEA4 has been associated with shorter OS.[11] Demethylation of CTAs serving as tumor associated antigens might be an opportunity to increase anti-tumor T cell responses.

Currently, several clinical trials are being conducted with DAC as an adjunct to ICB (NCT03019003, NCT03467178, NCT05317000).

Systemic treatment with DAC would also impact immune cells. Its impact on the adaptive immune response has not been characterized comprehensively yet. Notably, several ICM are known to be primarily regulated by methylation of upstream promotor sequences.[12,13] Recent evidence indicates a central role for DNA methylation in entrenchment of exhaustion programs and the subsequent failure of ICB-mediated rejuvenation.[14,15] In addition, low dose Decitabine was shown to be effective as an adjuvant in oncolytic viruses and provide prolonged resistance to exhaustion in DAC preincubated CAR-T cells.[16,17] Thus, DAC treatment can be expected to have impact on lymphocyte differentiation and effector function. An important subgroup of T cells is expressing CD39, which is enriched in exhausted, tumor-reactive T-cells.[18,19] In addition, CD39+ CD4 T cells have been shown to harbor FoxP3+ T cells as well.[20]

To investigate a future role of low-dose DAC for cancer-immunotherapy,[21] in this study we provide insights into proliferation dynamics and expression of cancer-testis antigens in HNSCC cell lines. Additionally, effects of DAC on PBMC of OPSCC from patients and healthy donors on the expression of twelve ICM (PD-1, CTLA4, BTLA, CD137, CD27, GITR, LAG3, OX40, TIM-3, VISTA, ICOS, TIGIT) with a focus on CD39+ T cells were studied.

## Materials and Methods

### Patients

Samples from patients with newly diagnosed locoregionally advanced OPSCC seen at the Department Otorhinolaryngology, Head and Neck Surgery of Ulm University Medical Center from 2014 to 2021 were collected with informed consent as 50ml citrate buffered blood before treatment initiation. 24 healthy control samples were also collected with informed consent. Clinicopathological data is shown in Table 1. HPV status was determined using multiplex PCR and Sanger sequencing [22] and p16^INK4a^ immunohistochemistry (Clone 1D7D2, ThermoFisher, Carlsbad, USA).

**Table 1:** Clinicopathological Characteristics of healthy donors and patients with oropharyngeal squamous cell carcinoma.

Parameter	OPSCC Patients (ICM) n=22			NC control Group (ICM) n=24	p-Value (OPSCC vs NC)	OPSCC Patients (Apoptosis) n=6 (4x HPV-2x HPV+)	NC (Apoptosis) n=4	NC (CFSE) n=4
	HPV positive n=12	HPV negative n=10	p-Value					
Mean age (range)	59.1 (46-69)	60.6 (53-70)	0.884	49.5 (24-72)	0.033	58.5 (53-64)	41.8 (25-65)	54.5 (48-62)
Sex Female Male	5 (41.7 %) 7 (58.3 %)	0 (0 %) 10 (100 %)	0.030	1x N/A 9 14	0.194	1 (16.7 %) 5 (83.3 %)	1 (25 %) 3 (75 %)	0 (0 %) 4(100 %)
T status 1 2 3 4	2 (16.7 %) 6 (50 %) 0 (0 %) 4 (33.3 %)	2 (20 %) 2 (20 %) 4 (40 %) 2 (20 %)				2 (33.3 %) 3 (50 %) 1 (16.7 %) 0 (0 %)		
HPV- St. DNA p16	11 (91.7 %) 11 (91.7 %)					2 (33.3 %) 1 (16.7 %)		
Smoking Never >5PY	6 (50 %) 6 (50 %)	1 (10 %) 9 (90 %)				3 (50 %) 3 (50 %)	4 (100 %) 0 (0 %)	
Alcohol Never/Moderate High	10 (83.3 %) 2 (16.7 %)	4 (40 %) 6 (60 %)				4 (66.7 %) 2 (33.3 %)	4 (100 %) 0 (0 %)	

Clinicopathological parameters for patients with oropharyngeal carcinoma (OPSCC) and healthy control (non-cancer/NC) lymphocyte donors. Statistical comparison of Age and sex category was performed by Mann-Whitney-Test and one-sided Fishers exact test, respectively. Alcohol consumption was classified as heavy, exceeding 4 Standard Drinks [39] per day for men and 3SD/ for women.

### PBMC Isolation and Storage

Peripheral blood mononuclear cells (PBMC) were isolated by density gradient centrifugation, using Leukosep tubes (Greiner bio-one, Frickenhausen, Germany). Red blood cell (RBC) lysis was done with RBC Lysis Solution (MACS Miltenyi, Bergisch Gladbach, Germany). 5 × 10^6^ PBMCs were slowly frozen in RPMI1640 medium (ThermoFisher) with 20 % fetal bovine serum (FBS) (distributed by Bio&Sell, Feucht, Germany) and 10 % DMSO (Sigma Aldrich) at a density of 1 × 10^6^/ml viable cells. After slow freezing at -80°C, samples were stored in gaseous phase of a liquid nitrogen tank until use.

### PBMC Stimulation and Treatment

Thawed and washed PBMC were resuspended in RPMI1640 medium with 10 % FBS and 1 % Penicillin/Streptomycin (PAN Biotech, Aidenbach, Germany). Trypan blue (Sigma-Aldrich) cell counting was done using a CellDropFL Cell Counter (DeNovix, Wilmington, USA). To achieve stimulation induced proliferation and S-Phase dependent DAC incorporation, ImmunoCult Human CD3/CD28 T-cell activator (Stemcell Technologies, Vancouver, Canada) was used at 25µl stock-solution per ml, rhIL-4 (Sartorius CellGenix, Freiburg, Germany) at 2ng/ml, and rhIL-2 (PeproTech, Hamburg, Germany) at 150IU/ml, subsequently plated in 12-well-suspension-plates (Sarstedt, Nümbrecht, Germany) at 2 × 10^6^ cells in 2ml per well and incubated at 37.0°C and 5 % atmospheric CO2. The stimulation and treatment regimens were used for all assays involving isolated PBMC's. On day 1, Decitabine (DAC, Selleckchem, Munich, Germany) or DMSO was added to each well. On day 3, 1ml of RPMI1640 medium, supplemented with IL-2, IL-4 and DAC or DMSO, was added to each well to achieve day 1 concentrations. The experimental workflow is depicted in **Supplementary Fig. 1**.

### Flow Cytometry and Gating

1 × 10^6^ cells/tube were washed and preincubated with human TruStain FcX Fc-blocking solution (422302, Biolegend, SanDiego, USA), followed by detection antibodies, and incubated for 30 min in the dark at room temperature. Tubes were washed, centrifuged, resuspended in 350µl PBS containing 5 % MACS bovine serum albumin (BSA) (130-091-376, Miltenyi Biotec, Bergisch Gladbach, Germany) and analyzed on a Gallios Flow Cytometer (PN775016, Beckman Coulter, Brea, USA). Analysis was performed in Kaluza Analysis software (A82959, Beckman Coulter). Compensation matrices were generated using beads (552843, BD Biosciences, Franklin Lakes, USA) or cultivated lymphocytes for apoptosis and proliferation assays. A system check, using Flow-Check Pro Fluorospheres (A53493, Beckman Coulter), was performed with every start up. Flow rate was adjusted to 1000 events per second. Doublet events and the first 30 seconds were excluded. Gating strategies are shown in **Supplementary Fig. 2A**. For non-dichotomous cell populations, cut-off values were determined using full-minus-one (FMO) controls and the corresponding isotype antibody with a false positive percentage of 0.5 % gated (**Supplementary Fig. 2B**). Exclusion of autofluorescent, non-viable cells[23] is shown in **Supplementary Fig. 2C**.

### Proliferation Assay

To investigate proliferation, a Carboxyfluorescein-Succinimidylester (CFSE) assay was used (C34554, Thermo Fisher). The recommended staining concentration of CFSE was lowered to 1µM. Cells were preincubated with CFSE and cultivated and treated as described in 2.3. Flow cytometric acquisition was performed on seeding day 0 as well as days 3 and 6. Non-viable cells were excluded by 7-Aminoactinomycin (7-AAD) stain. Division indices were calculated as previously described[24]. **Supplementary Fig. 5** depicts the gating of division generations.

### Apoptosis Assay

Cells were harvested, washed, and stained with detection-antibodies. They were resuspended with Annexin V Binding Buffer and subsequently stained with Fluorescein isothiocyanate (FITC)-labeled Annexin V and 7-AAD. After 10 minutes of incubation, flow cytometric acquisition started immediately. An unstained sample was acquired during incubation time of 7AAD and Annexin V.

### Immune Checkpoint Molecule Assessment

Complete staining panels with shared backbone markers as well as ICM detection antibodies are listed in **Supplementary Table 1**. To account for different emission spectra of tandem-fluorophores (PE-Cy5 in panel 1 and 2, PerCP-Cy5.5 in panel 3, PerCP eFluor 710 in panel 4), the bandpass-filter was adjusted to 675nm for panels 1 and 2 and 695nm for panels 3 and 4.

### Cell line treatment

Six HNSCC cell lines, three HPV positive (UD-SCC-2, RRID:CVCL_E325; UM-SCC-47, RRID:CVCL_7759; UPCI-SCC-090, RRID:CVCL_1899) and three HPV negative (UD-SCC-1, RRID:CVCL_E324; UD-SCC-4, RRID:CVCL_E327; UD-SCC-5, RRID:CVCL_L548), were cultivated in 175cm^2^ flasks containing DMEM without pyruvate (GIBCO, Thermo Fisher), 10 % FBS, 1 % ZellShield antibiotic mix (13-0050, Minerva Biolabs, Berlin, Germany) as well as 1 % non-essential amino acids (GIBCO). Cell splitting at 70-80 % confluency was performed, using 10ml Trypsin-EDTA solution (PAN Biotech).

Experiments were performed with short tandem repeat (STR) profiling authenticated vials of cell lines, frozen after identity authentication. All experiments were performed with PCR-tested mycoplasma-free cells. The cell lines were kindly provided by Prof. Dr. Thomas K. Hoffmann. Detailed cell line information and references are listed in **Supplementary Table 2**. Treatment was facilitated at the same timepoints as PBMC treatment with 1µM DAC or DMSO as control and subsequent reseeding was performed on day 6.

### MTT assay

Treated cell lines were assessed for metabolic activity using a Vybrant MTT Cell Proliferation Assay Kit (V13154, Invitrogen, Thermo Fisher) according to manufacturer's instruction. DAC treated cells were incubated in 96-well plates (Nunclon Delta, Thermo Fisher) at 10^5^ cells per well and absorbance was measured after 8 hours using an Infinite 200 Pro (Tecan, Männedorf, Switzerland). Results were normalized to the respective control group. Three independent experiments with 6 replicates have been performed.

### Cell line proliferation

Treated cells were reseeded in 25cm^2^ flasks at 10^6^ cells. Every 24 hours automated trypan blue cell counting was performed up to 96 hours after reseeding. Three independent experiments with duplicates have been performed.

### DNA & RNA isolation

Cell pellets were snap frozen and stored at -80°C. DNA and RNA isolation was performed using the AllPrep DNA/RNA Mini kit (80284, Qiagen, Venlo, Netherlands) according to manufacturer's instructions.

### cDNA reverse transcription and qRT-PCR of CTAs

Following cDNA preparation by QuantiNova Reverse Transcription Kit (205413, Qiagen) qRT-PCR was performed on a Lightcycler 96 (Roche, Basel, Switzerland). SYBR green (204143, Qiagen) was used as calibrated dye. CTA transcription was assessed with Glycerinaldehyd-3-phosphat-Dehydrogenase (GAPDH) as reference and commercially available human testis RNA as positive control (HR-401, Zyagen, San Diego, USA). CTAs and corresponding primer sequences are shown in **Supplementary Table 3**. The experiments were performed in triplicates in three independent experiments.

### Bisulfite DNA conversion and methylation pyrosequencing

Genomic 5-methylcytosine was converted using the Epitect Bisulfite Conversion Kit (59104, Qiagen) according to manufacturer's instructions. Multiplication of the regions of interest was performed using the PyroMark PCR Kit (Qiagen). High and low methylated human bisulfite converted DNA (EpigenDX, Hopkinton, USA), genomic DNA as well as only water were employed as controls. PyroMark primer sequences for the regions of interest are shown in **Supplementary Table 4**. Promotor regions were chosen according to the Eucaryotic promotor database (Dreos et al. 2017). PCR products with sufficient quality were prepared on a VacuumPrep workstation (Qiagen) and forwarded to sequencing on a PyroMark Q96 ID (Qiagen) platform, according to manufacturer's instructions. Analysis was performed in PyroMark sequencing software (Qiagen). Experiments were performed in triplicates in three independent experiments.

### Statistical analysis and visualization

Statistical analysis and visualization were performed using GraphPad Prism (version 9.3.1) (GraphPad Software, San Diego, USA) as well as R (version 4.1.2) using RStudio (version 2022.02.0) as integrated development environment. R packages are listed in **Supplementary Table 5**.

Friedemann test as well as Kruskall Wallis test was used to test for differences in ICM expression in paired and unpaired samples respectively. To account for multiple hypotheses tested, we applied a two-stage linear step-up procedure by Benjamini, Krieger and Yekutieli (Q=0.01). To test differences in treated cell lines, Mann-Whitney-U test was performed with p<0.05 considered significant.

## Results

### Clinicopathological Characteristics

Clinicopathological characteristics of OPSCC patients and PBMC donors as well as sample usage in respective experiments are shown in Table 1. There was a significant age disparity between NC and OPSCC, with a lower mean age in the NC group. HPV positive and negative individuals showed differences in sex proportion, smoking, and alcohol consumption.

### DAC treatment of head neck squamous cell carcinoma cell lines impairs proliferation and de-represses previously non expressed Cancer Testis Antigens

**Fig. 1:**
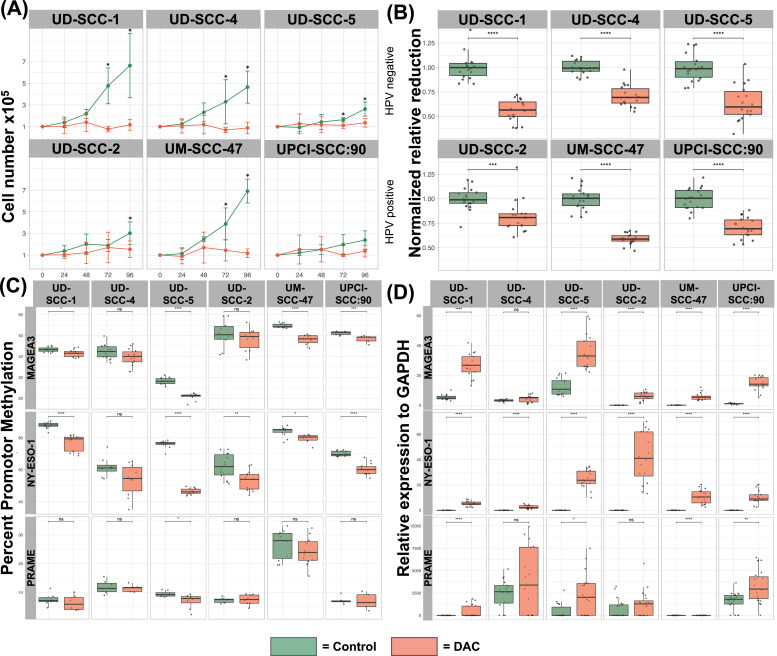
**DAC Treatment of HNSCC Cell lines**. Six HNSCC cell lines were treated during culture on days 1 and 3 following reseeding with 1µM DAC and DMSO as control. UD-SCC-1, UD-SCC-4 and UD-SCC-5 are derived from HPV- OPSCC while UD-SCC-2, UM-SCC-47 and UPCI-SCC-90 are derived from HPV+ OPSCC. DAC treated samples are depicted in red and the DMSO controls in green. **(A)** Proliferative capability following reseeding on day 6 of treatment was shown to be severely impaired in all cell lines. Except for UPCI-SCC-90 all control samples started regrowing after 96 hours. Three independent experiments with duplicates were performed. Mann-Whitney-U test was used for comparison. p-values < 0.05 were considered significant. **(B)** Impact of DAC on metabolic activity of cancer cells was evaluated in an MTT Assay. Metabolic activity of all treated cell lines was reduced significantly. Three independent experiments were conducted with six replicates each. Mann-Whitney-U test was used for comparison. Error bars represent SD, p-values < 0.05 were considered significant. **(C)** To verify the proposed demethylation effect, we assessed promotor methylation of three cancer testis antigens by Bisulfite-Pyrosequencing. Except for UD-SCC-4, in every cell line, significant promotor demethylation was observed. Three independent experiments with triplicates were performed. Mann-Whitney-U test was used for comparison. p-values < 0.05 were considered significant. **(D)** To evaluate the transcriptional impact of demethylation we conducted qRT-PCR of the corresponding CTAs. For every significantly demethylated promotor sequence, we observed significantly upregulated transcription. Three independent experiments with triplicates were performed. Mann-Whitney-U test was used for comparison. p-values < 0.05 were considered significant.

To investigate the proposed demethylation effect of the PBMC treatment regime on cancer cells, we evaluated proliferation as well as expression of three CTAs and their corresponding promoter methylation in six head and neck squamous cell carcinoma (HCSCC) cell lines post-treatment. Figs. 1**A and** 1**B** show significantly abolished proliferation and metabolic activity 96 hours after reseeding in HPV positive as well as negative cell lines. One exception was UPCI-SCC:90, with slow proliferation in control cells. Next, we analyzed DAC mediated de-repression of immunogenic antigens in HNSCC cell lines, with a focus on three CTA, Melanoma-Antigen A3 (MAGEA3), New York Esophageal Squamous Cell Carcinoma-1 (NY-ESO-1), as well as Preferentially Expressed Antigen in Melanoma (PRAME) and their corresponding promotor sequences. Fig. 1**C** shows mean promotor methylation percentage, assessed by bisulfite sequencing. In every cell line, methylation of at least one promotor was reduced, comparing DAC to DMSO treatment, except for UD-SCC-4 where a trend for demethylation in MAGEA3 as well as NY-ESO-1 did not reach significance. qRT-PCR was then performed to analyze the transcription of the downstream coding regions, resulting in a significant increase in transcription following DAC treatment, as shown in Fig. 1**D**. Notably, NY-ESO-1 was de-novo expressed in all cell lines upon DAC treatment. MAGEA3 was de-novo expressed in all HPV+ cell lines and increased in all HPV- cell lines upon treatment except for UD-SCC-4. 5 cases of increased transcription did not match significant demethylation, with a trend towards reduced methylation observed in 3 of these cases. Conversely, significant promoter demethylation resulted in a significant increase in transcription in all 10 cases. In conclusion, DAC treatment led to impaired proliferation, promoter demethylation, and expression of CTA antigens in all cell lines.

### DAC treatment of lymphocytes dose dependently impairs proliferation with predominance in CD39 positive T cells and B cells

**Fig. 2:**
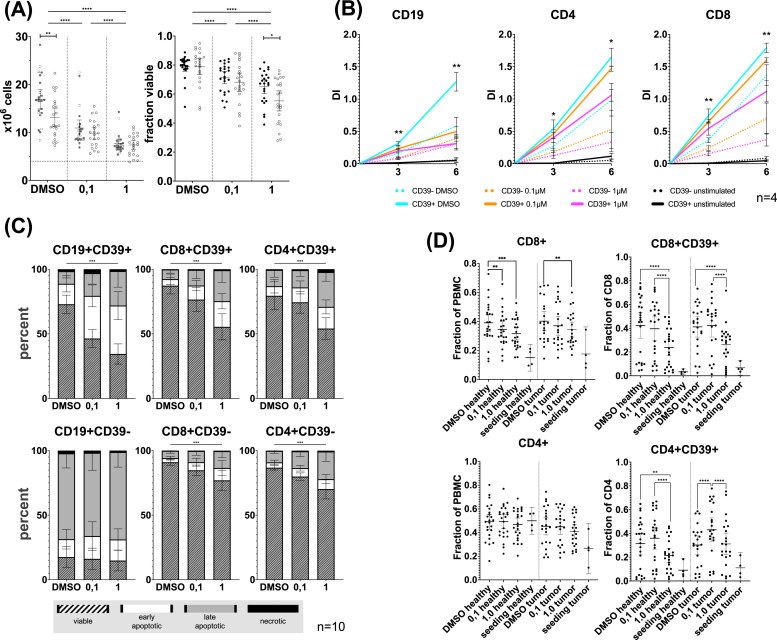
**Proliferation, Viability, and fractions of subpopulations after six-day cultivation**. Donor lymphocytes were cultivated in vitro for 6 days with decitabine (Dacogen) treatment at 0,1µM and 1µM and DMSO as control. **(A)** After cultivation the number and viability of the donor lymphocytes were measured by automated trypan blue cell counting. The individual values for healthy donors and tumor patients are depicted as black dots or circles, respectively, in scatter plots. DAC treatment resulted in significant reduction of viability and cell number. In OPSCC samples we observed significantly reduced cell numbers in DMSO and reduced viability after 1µM DAC treatment. **(B)** A CFSE assay was conducted on four normal control (NC) samples. The Division Indices (DI) were calculated based on the decrease in fluorescence intensity. A two-sided, paired t-test was used to evaluate the differences in the DI between unstimulated and 1 µM DAC treated, CD3CD28 stimulated samples in the CD39- fraction, which represents population with the lowest proliferation. Compared to unstimulated samples, all subfractions in all stimulated treatment regimens achieved significant proliferation, with ** = p<0.01, *=p<0.05**(C)** Bar plots illustrate results of an AnnexinV/7AAD apoptosis assay of ten cultivated samples. Percentages of viable, early apoptotic, late apoptotic, and necrotic cells are stacked. The differences in the viable fraction were tested using Friedemann tests and corrected for multiple testing. Treatment with 1 µM DAC, but not 0.1 µM DAC, resulted in significantly reduced viability in all subfractions except CD19+CD39- cells, which had distinctly low viability. No significant difference in viability was observed in CD4+ and CD8+ cells based on CD39 status. **(D)** ICM expression was measured on different subsets via flow cytometry. We observed significant decrease of CD8+ but not CD4+ cells. DAC treatment had significant impact on the fraction of CD39 positive cells in both subpopulations. Lines are drawn at mean with whiskers spanning the 95 % confidence interval. Statistical analysis for treatment effects in NC and OPSCC lymphocytes were performed using a Friedemann test, analysis for difference between NC and OPSCC lymphocytes in each treatment group was performed with Kruskall Wallis test. The two-stage linear step-up procedure of Benjamini, Krieger and Yekutieli was used for correction of multiple testing (Q = 0.01). Significant results are marked with asterisks ****=p<0.0001 *** = p<0.001 ** = p<0.01.

Six-day culture of PBMC treated with DAC revealed dose dependent reduction in cell number and viability, as was seen in cancer cell lines. (Fig. 2**A**) Reduced viability was pronounced in OPSCC patients’ samples. For DAC to exert it's demethylating effect, proliferation of treated cells is a prerequisite. A CFSE-assay could validate significant proliferation in all subsets, shown in Fig. 2**B.** DAC exerted a dose dependent decrease in proliferation. CD39 expression was associated with increased proliferation, but also higher rates of apoptosis in T-cells in an AnnexinV/ 7-AAD assay, displayed in Fig. 2**C**. Fig. 2**D** depicts DACs effects on T cell subset composition, revealing significantly lower CD8 but not CD4 T cells in all samples. CD39 expression exhibited notable heterogeneity and nonlinear DAC effects with general reduction in 1µM but increased expression in the 0,1µM DAC treated subset of OPSCC patients CD4 T cells.

### DAC treatment results in pronounced alterations of the ICM repertoire, influenced by donor type and CD39 expression for some but not all altered ICMs

**Fig. 3:**
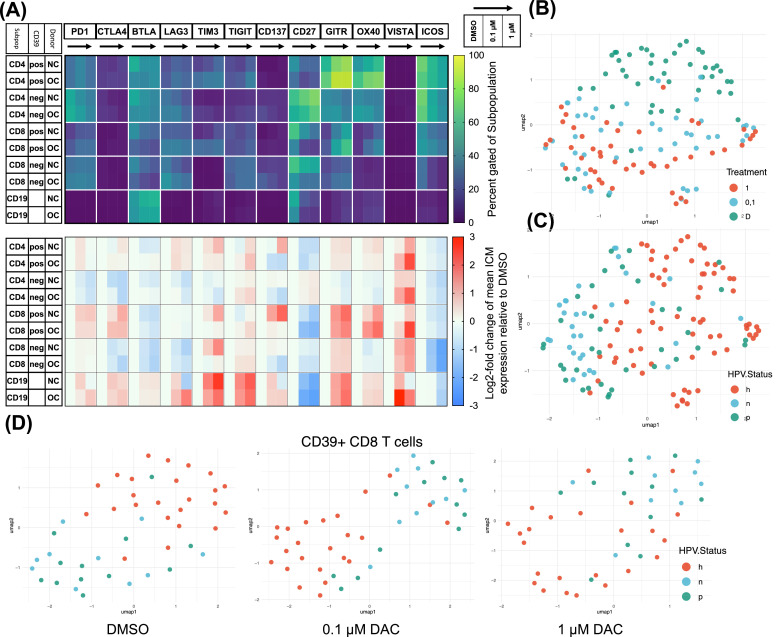
**Treatment effect on ICM expression and differences in donor type**. Donor lymphocytes were cultivated in vitro for 6 days with decitabine (Dacogen) treatment at 0,1µM and 1µM and DMSO as control. Subsequently cells were assessed flow cytometrically for expression of twelve ICMs on five subpopulations. **(A)** The shown heatmaps display mean gated-positive percentage at the top and log-2-fold change relative to DMSO control at the lower panel. Each checkpoint is displayed with the treatment groups sorted from left to right, starting at DMSO, indicated by the black arrow. The five covered subpopulations are subdivided by donor type into NC (h) and OPSCC (t). We observed manifold expression changes upon DAC treatment, most prominently, increased expression of GITR and OX40 in CD39+ T-cells. ICOS and CD27 expression was significantly decreased in all subpopulations, except for CD27 in CD4+CD39- cells. **(B)** UMAP of all samples based on expression values of all ICMs in all subpopulations is shown. Donor type is depicted as color code with “h” resembling healthy, “p” HPV-positive and “n” HPV-negative. Treatment groups are labeled with “D” for DMSO, “0,1″ for 0.1µM and “1″ for 1µM decitabine (Dacogen) treatment. We observed clear treatment-dependent embedding with a gradual dose dependence. (Hyperparameters: spread 0.8, n_epochs: 1000, min_dist: 0.1, n_neighbors: 15) **(C)** The same UMAP highlights distinct clustering dependent on donor type with HPV negative samples being mapped more closely, indicating a more homogeneous sample collective. **(D)** Three treatment-wise UMAPs of the CD39+ CD8 T cell subpopulation reveal similar mapping patterns dependent on donor type with HPV positive samples tending to be more intertwined with healthy donor samples. While HPV negative samples were embedded more closely. (Hyperparameters: Spread 1, n_epochs: 1000, min_dist: 0.1, n_neighbors: 13).

Several ICMs are known to be regulated by promotor methylation. We performed Flow Cytometric ICM profiling following DAC treatment. Fig. 3**A** displays the mean expression percentage of each ICM and log2-fold change dynamics of the corresponding ICM compared to DMSO controls. We observed divergent ICM expression patterns in samples derived from OPSCC patients, compared to NC. We utilized uniform manifold approximation and projection (UMAP) to create a representation of sample similarity based on ICM expression and cell fraction data. The resulting UMAP visualization shown in Figs. 3**B and** 3**C**, shows the differentiation between the three treatment conditions. Embedding of DMSO and 1µM with the 0.1µM DAC treatment group interposed, resembles a gradual treatment effect. Fig. 3**C** reveals additional distinction between OPSCC and NC samples irrespective of the treatment type. Interestingly HPV negative OPSCC samples show closer embedding, indicative of a more heterogeneous collective while HPV positive samples appear closer to NC samples. This tendency was observed for CD39+ CD8 T cells in particular (Fig. 3**D)**. Further comparisons of individual ICM expression trends by HPV status of OPSCC patients are shown in **Supplementary Fig. 3.**

### DAC's effects are influenced by CD39 expression in T cells for OX40, GITR, CD27 but not ICOS

**Fig. 4:**
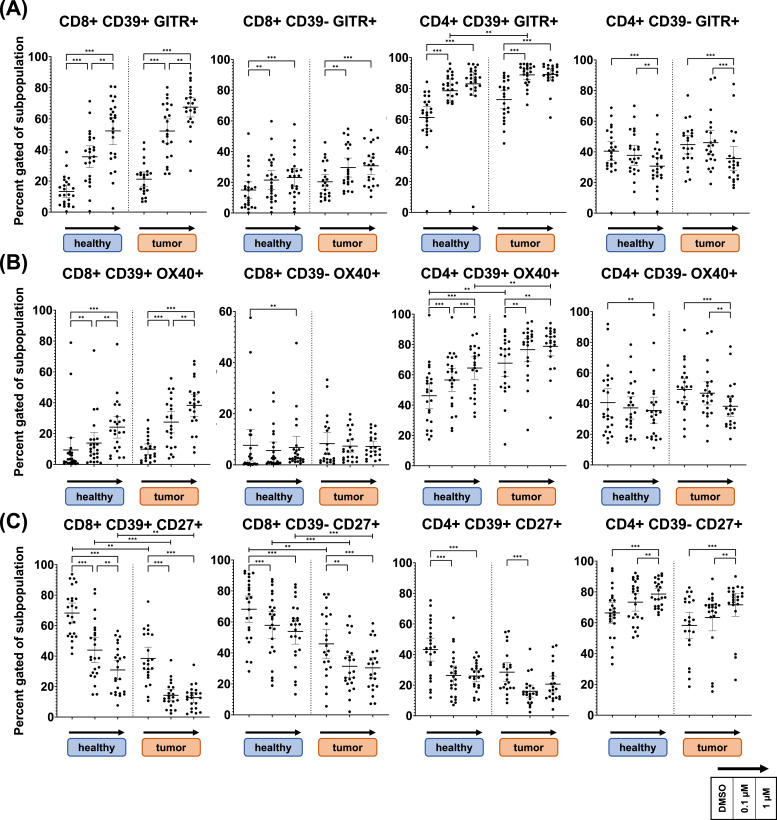
**GITR, OX40 and CD27 expression changes, influence of CD39 and donor type**. Donor lymphocytes were cultivated in vitro for 6 days with decitabine (Dacogen) treatment at 0,1µM and 1µM and DMSO as control. Subsequently cells were assessed flow cytometrically for expression of twelve ICMs. Fig. 4 shows expression of co-stimulatory ICMs GITR, OX40 and CD27 in CD4 and CD8 T cells. As shown on panel **A**, GITR shows higher expression in CD4+ cells and significantly higher expression in OPSCC derived CD39+ CD4 T cells. Except for CD39- CD4 T cells, GITR significantly increased in all subpopulations. The same pattern was observed for OX40 except for CD39- CD8 T cells, showing only marginal alteration in expression. Shown in panel **B**. Conversely, CD8 T cells of OPSCC patients, expression of CD27 was significantly lower expressed, as seen in panel **C**. This difference was maintained in all treatment groups. In addition, DAC treatment resulted in significant decrease of CD27 expression in all subsets, only opposed by CD4+CD39- cells with a significant increase. Lines are drawn at mean with whiskers spanning the 95 % confidence interval. Statistical analysis for treatment effects in NC and OPSCC lymphocytes were performed using a Friedemann test, analysis for difference between NC and OPSCC lymphocytes in each treatment group was performed with Kruskall Wallis test. The two-stage linear step-up procedure of Benjamini, Krieger and Yekutieli was used for correction of multiple testing (Q = 0.01). Significant results are marked with asterisks ****=p<0.0001 *** = p<0.001 ** = p<0.01.

Flow cytometric analysis of ICM expression reveals subpopulation-spanning DAC effects with dependence on CD39 expression. Fig. 4 displays expression of co-stimulatory ICMs GITR, OX40 and CD27 after six-day treatment. As seen in row **A**, GITR expression was strongly increased in the CD39+ fraction of DAC treated CD4 and CD8 T cells. CD4 cells show a higher baseline expression of GITR and OX40 maintained in DAC treated samples. Comparison of OPSCC (tumor) to NC (healthy) samples, reveals significantly higher OX40 expression in CD39+ CD4 T cells derived from OPSCC patients (Row **B**). CD39- subsets show less pronounced increase of GITR and modest OX40 expression in CD8 T cells and significantly decreased expression in CD4 T cells. Influence of CD39 and donor type was observed for CD27 as well (**Row C**). In CD8 T cells, CD27 was expressed significantly lower in OPSCC samples compared to NC and reduced by DAC in both groups. Difference was maintained throughout treatment. Contrasting dynamics in response to DAC treatment were observed for CD4 T cells with significant reduction of CD27 in CD39+ and significant increase in CD39- subsets.

**Fig. 5:**
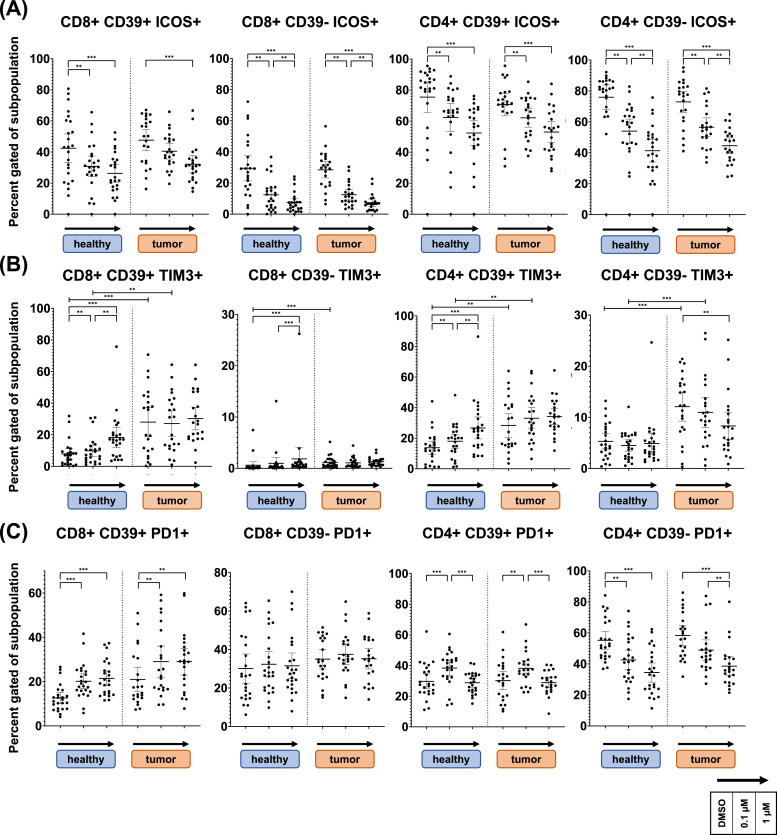
**ICOS, TIM3 and PD1 expression changes, influence of CD39 and donor type**. Donor lymphocytes were cultivated in vitro for 6 days with decitabine (Dacogen) treatment at 0,1µM and 1µM and DMSO as control. Subsequently cells were assessed flow cytometrically for expression of twelve ICMs. Fig. 6 shows expression of co-stimulatory ICM ICOS as well as co-inhibitory ICMs TIM3 and PD1 in CD4 and CD8 T cells. Panel **A** shows, that ICOS was globally expressed higher in CD4 than in CD8 T cells and significantly decreased in response to DAC treatment in all subpopulations. Interestingly, baseline TIM3 expression was significantly higher in all subpopulations of OPSCC derived samples, as shown in panel **B**. In CD39+ CD4 and CD8 T cells, TIM3 expression significantly increased in NC samples, to reach OPSCC expression levels at 1µM DAC. Contrary, elevated expression in CD39- CD4 T cells of OPSCC patients significantly decreased to reach NC levels. Panel **C** shows expression percentage of PD1. Its expression was significantly increased in CD39+ CD8 T cells. Opposingly, PD1 expression was significantly reduced in CD39- CD4 T cells. In CD39+ CD4 T cells an inverse u shaped response was observed. Lines are drawn at mean with whiskers spanning the 95 % confidence interval. Statistical analysis for treatment effects in NC and OPSCC lymphocytes were performed using a Friedemann test, analysis for difference between NC and OPSCC lymphocytes in each treatment group was performed with Kruskall Wallis test. The two-stage linear step-up procedure of Benjamini, Krieger and Yekutieli was used for correction of multiple testing (Q = 0.01). Significant results are marked with asterisks ****=p<0.0001 *** = p<0.001 ** = p<0.01.

Fig. 5 displays expression of co-stimulatory ICM ICOS as well as co-inhibitory ICM TIM3 and PD1. For ICOS, DAC led to a significant, dose dependent decrease in expression throughout all subsets (**Row A**). A CD39 dependent pattern could be observed for TIM3 and PD1. CD39- CD4 T cells decreased PD1 expression upon DAC treatment, levels were unaltered in CD8 T cells (**Row C**). For CD39+ T cells, PD1 was significantly increased in CD8 T cells while an inverse U shaped response was observed in CD4 T cells. For TIM3 we observed prominent donor influence (**Row B**). Notably CD39- CD8 T cells did non express TIM3. In CD39+ T cell subsets, TIM3 expression was significantly higher in OPSCC than in NC samples and did not change upon DAC treatment. In CD39- CD4 T cells of OPSCC samples, TIM3 decreased to reach NC levels upon DAC treatment. Additional donor type specific effects were observed for inhibitory ICMs BTLA and TIGIT, as shown in **Supplementary Fig. 4**.

## Discussion

In this study, we assess the complex alterations on HNSCC cells and immune cells following demethylating treatment. Promoting the expression of cancer antigens in cancer cells may serve as an attractive strategy to initiate a broader anti-tumor response. At the same time, alterations of ICM expression on immune cells, pose an opportunity in the light of rapid advances in therapeutic ICM targeting.

In all HNSCC cell lines DAC treatment resulted in a methylation-dependent change in cancer testis antigen expression. This was particularly interesting for HPV-positive cell lines, which were all negative for expression of MAGEA3 and NY-ESO-1 but showed increased CTA expression after DAC treatment. Vaccines against MAGEA3, NY-ESO-1 and PRAME can elicit cancer-specific T cell responses [[25], [26], [27], [28], [29]] implying potentially increased immunogenicity of HNSCC in response to DAC treatment. Previous work found expression of CTAs to be associated with unfavorable prognosis in HNSCC patients.[9] In this work, we did not observe a growth advantage accompanying CTA expression in response to DAC treatment, but severely impaired proliferative capability in all treated cell lines.

In the immune cell compartment, we observed striking effects on co-stimulatory ICM, such as OX40, GITR, CD27, and ICOS, as well as co-inhibitory ICM, such as PD1, TIM3, BTLA, and TIGIT. We noted polarizing effects on CD4 and CD8 T cells depending on CD39 expression. Overall, the effects of DAC in this study can be categorized into three groups: global effects that are similar across all subsets (ICOS), CD39-dependent effects that are partly influenced by CD4 or CD8 polarization (GITR, OX40, CD27), and donor-type specific effects (OPSCC vs. NC) that primarily affect exhaustion-associated inhibitory ICMs (TIGIT, TIM3, BTLA).

The observed effect of distinct responses to DAC in CD4 and CD8 T cells are consistent with recent findings, indicating different impact of impaired de novo methylation during activation: In CD8 T cells, hypomethylating agents were shown to induce a heightened activation state and cytotoxic capability.[30] In CD4+ T cells, impaired methylation was shown to result in increased lineage plasticity.[31] In CD8 and CD4 T cells, CD39+ has been reported as an activation marker,[32] identifying antigen-experienced cells in vivo and indicating a higher level of DAC incorporation and demethylation. This is consistent with our observation of pronounced expression changes in these immune cell subsets. Interestingly, CD27 displayed opposing dynamics compared to GITR and OX40 expression in CD8+ T cells, but only minor changes in CD39+ CD4 T cells, whereas in CD39- CD4+ T cells CD27 expression increased dose dependently. The loss of CD27 has been reported to be a characteristic of an induced effector phenotype of CD4+ T-cells and CD8+ T-cells.[33,34] The significantly lower CD27 expression in OPSCC patients compared to NC further supports our hypothesis of a globally altered immune cell pool. A lower abundance of CD27 in the peripheral blood could indicate a higher fraction of differentiated memory T cells in response to chronic antigenic stimulation. CD39+ CD8 T cells have previously been shown to also express elevated levels of TIM3, which is in accordance with our own observations.[35] Further, co-expression of TIM3 and CD39 was proposed as a marker of exhaustion like T cells.[18,36] Yet, CD39 was shown to identify tumor-reactive, phenotypically exhausted infiltrating CD8 T lymphocytes, still capable of cytotoxic function and recruitable by ICB.[37] Although these observations do not allow transfer to in vitro stimulated lymphocytes, we observed TIM3 to be expressed significantly higher in CD39+ CD8 T cells of OPSCC patients.

Strikingly we observed ICOS to be significantly less expressed in all subpopulations of healthy donors’ and tumor patients’ samples in response to DAC treatment, suggestive of a separate lineage independent mechanism. ICOS is known to be preferentially expressed in Th2 polarized helper cells, and less in the Th1 lineage.[38] In accordance to that is the observation of differentiation skewed towards Th1 with DNMT-inhibitor (DNMTi) treatment during activation.[39] In naïve T-cells STAT3 is required for ICOS expression,[40] which was shown to be downregulated in AML- as well as hepatocellular carcinoma cell lines, treated with DAC.[41,42] This could be explained by upregulation of members of Suppressor of Cytokine Signaling Proteins (SOCS), known to be silenced by DNA-methylation and upregulated by DAC treatment.[43]

Despite the treatment being performed in vitro, our observations showed pronounced differences in responses between cells from OPSCC patients and healthy donors, suggesting a rather stable immune cell dysfunction in OPSCC patients. This could be also reflected in the predominant increase of TIGIT expression in OPSCC samples in response to treatment.[44] Furthermore, samples from HPV+ patients appeared more similar to those of healthy donors in a dimensionality reduction analysis. However, samples from HPV- patients appeared to be more homogeneous, which may be due to common carcinogenesis and a narrower age range among these patients.

The prerequisite for DAC-mediated demethylation is its incorporation during S-phase. Demethylation of methylated loci then occurs via passive dilution, while de novo methylation could be disrupted in a lineage-specific manner. The results from the CFSE assay indicate that this necessity was met in all subpopulations, with differences in magnitude. Both, HNSCC HPV-negative and -positive cell lines displayed reduced proliferative capacity, primarily in those cell lines with a higher baseline proliferation rate. Among the lymphocyte subpopulations a similar effect was observed. CD39+ subsets exhibited higher proliferation, which may also explain the pronounced effects on ICM expression in these cells. The observed changes in the frequency of CD39+ cells with DAC treatment suggest a potential selection effect. Recent studies have demonstrated, that CD39+ CD4 T cells are phenotypically exhausted and more susceptible to undergo apoptosis compared to CD39- cells.[45] This is consistent with our observation of a slightly elevated rate of apoptosis in the CD39+ subfractions. Increased proliferation of the CD39+ subsets may have contributed to increased DAC cytotoxicity and subsequent depletion. The notable increase in the CD39+ CD4 T cell fraction in samples treated with 0.1µM DAC is consistent with the activating effects of DAC, which may have been diminished by increased cytotoxicity at 1µM. This may also explain the inverse U-shaped expression of PD-1 in CD39+ CD4 T cells. While the CD39+ subfraction in CD4 T cells significantly overlaps with part of the FoxP3+ CD4 T cell subfraction, capable of suppressive function,[20,46] in vitro stimulation, and subsequent upregulation of CD39 does not sufficiently identify this subpopulation anymore, rather being representative of an activated state without increased FoxP3 expression.[45] Aligning the observed responses with known regulatory mechanisms is challenging. DAC is known to perturb various transcriptional programs, potentially resulting in increased lineage plasticity. The impact of DAC on DNA methylation and subsequently transcriptional regulation is complex. Cryptic transcription starting sites and intragenic promotors are often extensively methylated to reduce antisense transcription and alternative polyadenylation, for example.[47] Perturbation of this process was shown to mediate CD8+ stimulating effects in DAC-treated T cells by skewing the NFAT-1 transcription towards shorter, effector-promoting, isoforms.[48] This is in line with the observation that ERV sequences are employed in certain lineage-specific regulatory networks of immune cells.[49]

In summary, we could observe demethylation-mediated expression of CTAs in combination with a profound antiproliferative effect of DAC in HNSCC cell lines. At the same time, DAC treatment of PBMC, revealed distinct effects on ICM expression which were pronounced in CD39+ T cells, potentially enriched for antigen-experienced cells. Therefore, our results indicate synergistic effects by increasing CTA expression on cancer cells and preferable ICM expression patterns which may increase susceptibility to ICB for broader and durable anti-tumor immune responses.

## Conclusion

Effects of Decitabine (DAC) on immune cells are complex and have the potential to disturb differentiation programs in immune cells. The pronounced cytotoxicity at the 1 μM treatment dose implies more beneficial effects at much lower dosages. Our results suggest that the use of therapeutic GITR and OX40 agonism may further amplify the effects of DAC, promote T-cell survival, and provide therapeutic benefits.

## Ethics Statement

The study was conducted after approval of the local ethics committee of Ulm University with informed consent of participating patients and healthy controls (#222/13; #90/15).

## Funding

Simon Laban, Adrian Fehn, Jasmin Ezić and Thomas K. Hoffmann were part of the 10.13039/501100011633research training group GRK-2254 (HEIST, 288342734) funded by 10.13039/501100001659Deutsche Forschungsgemeinschaft (DFG, German Research Foundation). Simon Laban was funded by University of Ulm within the Clinician Scientist Program.

## CRediT authorship contribution statement

**Adrian Fehn:** Conceptualization, Data curation, Formal analysis, Investigation, Visualization, Writing – original draft, Writing – review & editing, Methodology, Project administration, Software. **Adrian von Witzleben:** Writing – review & editing. **Ayla Grages:** Writing – review & editing. **Tsima Abou Kors:** Conceptualization, Supervision, Writing – review & editing. **Jasmin Ezić:** Data curation, Formal analysis, Writing – review & editing, Investigation. **Annika C. Betzler:** Methodology, Writing – review & editing. **Cornelia Brunner:** Methodology, Supervision, Writing – review & editing. **Patrick J. Schuler:** Writing – review & editing, Methodology. **Marie-Nicole Theodoraki:** Writing – review & editing. **Thomas K. Hoffmann:** Resources, Writing – review & editing. **Simon Laban:** Conceptualization, Project administration, Resources, Supervision, Writing – review & editing.

## Declaration of competing interest

The authors declare the following financial interests/personal relationships which may be considered as potential competing interests:

Simon Laban: Advisory Boards: Merck Sharp & Dohme (MSD), Bristol Myers Squibb (BMS), Sanofi Genzyme, Astra Zeneca (AZ). Honoraria: MSD, BMS. Travel reimbursement: Merck Serono, Astra Zeneca. Thomas K Hoffmann: Advisory Boards: Merck Sharp & Dohme (MSD), Bristol Myers Squibb (BMS), Sanofi Genzyme.

## Appendix Supplementary materials

Image, application 1

Image, application 2
