# Supplementary material for: 5-Aza-2′-deoxycytidin (Decitabine) increases cancer-testis antigen expression in head and neck squamous cell carcinoma and modifies immune checkpoint expression, especially in CD39-positive CD8 and CD4 T cells
Source: Neoplasia. 2024 Nov 27;59:101086. doi: 10.1016/j.neo.2024.101086 (PMC11636331; doi:10.1016/j.neo.2024.101086)
Supplement: Supplementary file 1 [file mmc1.docx]

**Supplementary Figure 1.**

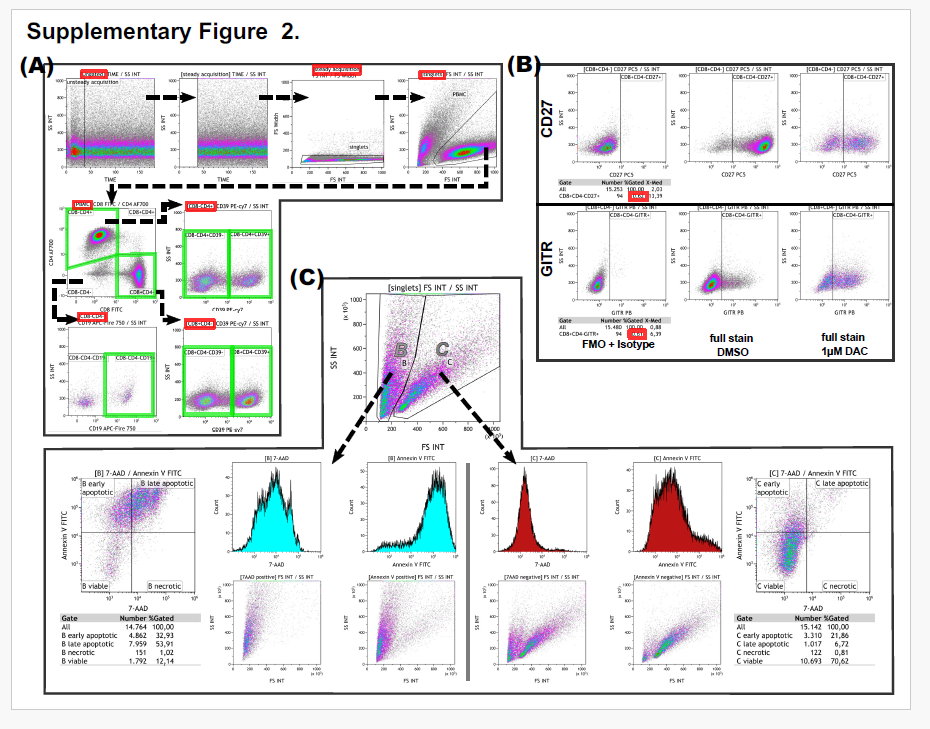


**Supplementary Figure 4.**


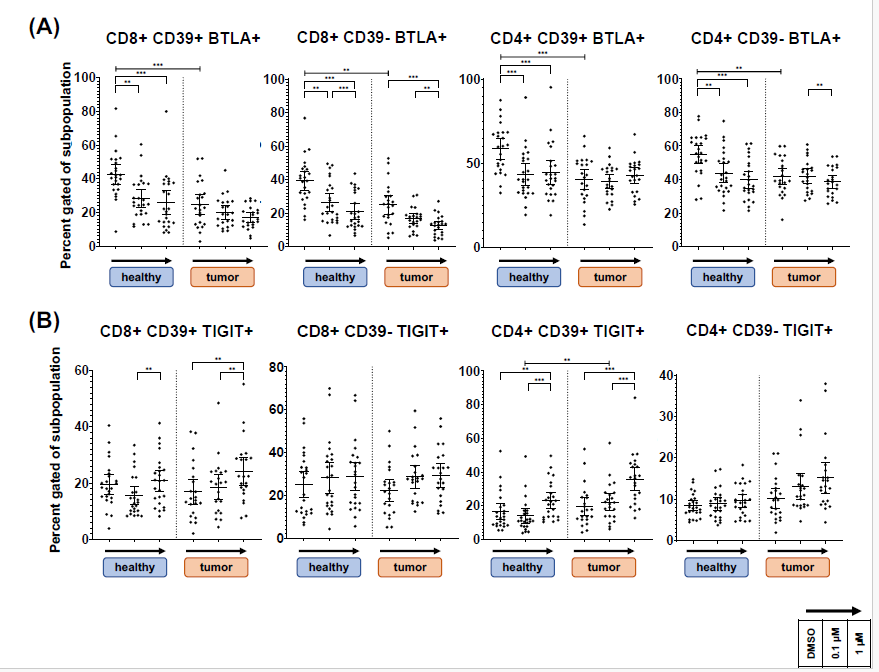


**Supplementary Figure 5.**


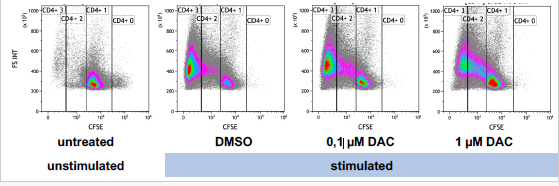


**Supplementary Figure 3.**
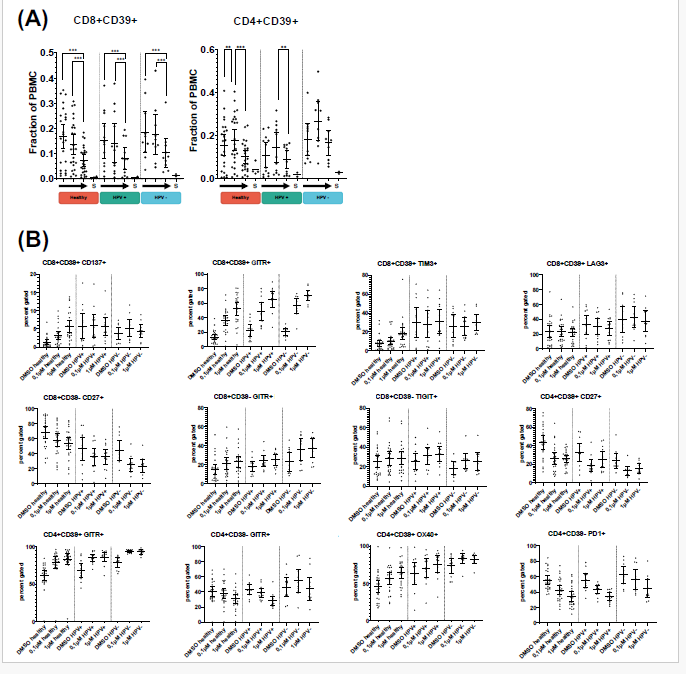


| **Name** | **HPV** | **Site of origin** | **Reference/Source** | **RRID** |
| --- | --- | --- | --- | --- |
| **UD-SCC-1** | **Negative** | **Oropharynx** | **(Balló et al., 1999)** | **CVCL_E324** |
| **UD-SCC-2** | **Positive** | **Hypopharynx** | **(Balló et al., 1999** | **CVCL_E325** |
| **UD-SCC-4** | **Negative** | **Oropharynx** | **(Balló et al., 1999)** | **CVCL_E327** |
| **UD-SCC-5** | **Negative** | **Larynx** | **(Hoffmann et al., 2008)** | **CVCL_L548** |
| **UM-SCC-47** | **Positive** | **Oral cavity** | **(Bradford et al., 2003)** | **CVCL_7759** |
| **UPCI-SCC-090** | **Positive** | **Oral cavity** | **(White et al., 2007)** | **CVCL_1899** |

**Supplementary Table 2.**

| **Gene** | **Direction** | **Sequence** |
| --- | --- | --- |
| **GAPDH** | **FW** | **CCATGGGGAAGGTGAAGGTC** |
|  | **RW** | **AGTGATGGCATGGACTGTGG** |
| **MAGEA3** | **FW** | **TCCTGTGATCTTCAGCAAAGCTT** |
|  | **RW** | **GGGTCCACTTCCATCAGCTC** |
| **CTAG1B** | **FW** | **TGCTTGAGTTCTACCTCGCCA** |
|  | **RW** | **TATGTTGCCGGACACAGTGAA** |
| **PRAME** | **FW** | **GTGCTCGTAGACCTGTTCCT** |
|  | **RW** | **CTGGCCCAGGTAAGGAGAAA** |

**Supplementary Table 3.**

| **Gene** | **Type** | **Sequence** |
| --- | --- | --- |
| **MAGEA3** | **FW** | **GGTAGTAGAGGTAGTATTGGATTATTTG** |
|  | **RW** | **ACCCCATCACCATCTTCA** |
|  | **Seq** | **CACTAACTTACCCATTAAAAATCA** |
| **CTAG1B** | **FW** | **GTTAGGGTTTTTTGGGTTATTAGTAT** |
|  | **RW** | **AACTTCCTACAACCTCTCTACC** |
|  | **Seq** | **CCCTAACCTTCTCTCTAA** |
| **PRAME** | **FW** | **GTTTTGTTTTTTTTATATTTAGGGTTGTT** |
|  | **RW** | **ACATACAATCCCTATTAACTCTAATACC** |
|  | **Seq** | **GTTAATATAAATTTGTTGATAGGT** |

**Supplementary Table 4.**

| **Package** | **Version** | **Reference** |
| --- | --- | --- |
| **Broom** | **0.7.2** | **(Robinson et al., 2020)** |
| **data.table** | **1.13.2** | **(Dowle & Srinivasan, 2020)** |
| **dplyr** | **1.0.2** | **(Wickham et al., 2021)** |
| **ggplot2** | **3.3.2** | **(Wickham, 2016)** |
| **readr** | **1.4.0** | **(Wickham & Hester, 2020)** |
| **tibble** | **3.0.4** | **(Müller & Wickham, 2021)** |
| **tidyr** | **1.1.2** | **(Wickham, 2021)** |
| **tidyverse** | **1.3.0** | **(Wickham et al., 2019)** |
| **umap** | **0.2.9.0** | **(Tomasz, 2020)** |

**Supplementary Table 5.**
