# Supplementary material for: 5-Aza-2′-deoxycytidin (Decitabine) increases cancer-testis antigen expression in head and neck squamous cell carcinoma and modifies immune checkpoint expression, especially in CD39-positive CD8 and CD4 T cells
Source: Neoplasia. 2024 Nov 27;59:101086. doi: 10.1016/j.neo.2024.101086 (PMC11636331; doi:10.1016/j.neo.2024.101086)
Supplement: Supplementary file 2 [file mmc2.docx]

**Supplementary Figure legends:**

**Supplementary Figure 1: Experimental workflow for lymphocyte treatment and flow-cytometric assessment**

Blood samples were acquired from patients diagnosed with oropharyngeal squamous cell carcinoma (OPSCC) and healthy donors (NC/HD). Density gradient isolation of PBMCs was performed. Cells were slowly frozen and stored in liquid nitrogen upon further cultivation. Cells were cultivated in RPMI medium for six days, stimulated with CD3/CD28 antibody as well as IL2 and IL4. On days 1 and 3 treatment with either DAC in two concentrations or DMSO as control was performed. Subsequent so harvesting on day 6 viability analysis and multi-color flow cytometric assessment of Immune Checkpoint expression as well as CFSE proliferation assay and an apoptosis assay was performed.

**Supplementary Figure 2: Gating and exclusion strategy**

**Panel A** shows the gating strategy of flow cytometric data. The first 30 seconds were dismissed due to instable acquisition. Doublet events were excluded by plot of forward scatter width values against integral values. Viable PBMCs were subsequently gated by morphologic criteria in the sideward scatter vs forward scatter plot. Different subpopulations were defined by abundance of subpopulations markers. **Panel B** exemplary describes cut-off determination for CD27 and GITR. Full minus controls and corresponding isotype antibodies were used to stain the control samples. Subsequently, cutoffs were chosen to include between 0,5 and 1% of events in the positive gate, resembling false positives. Adjunct to that, gating of fully stained and treated samples with the predetermined cutoffs is shown. **Panel C** covers the rationale behind the morphologic inclusion of events in gate C as mostly viable, non-auto-fluorescent cells. Virtually all 7AAD positive and the major fraction of highly AnnexinV positive events are in gate B. This indicates gate B to incorporate severely altered cells as well as non-staining debris whereas functional cells without debris are recorded in gate C.

**Supplementary Figure 3: Impact of HPV status on expression of immune checkpoint molecules**

PBMCs, isolated from healthy donors (NC) and patients diagnosed with oropharyngeal squamous cell carcinoma (OPSCC), were cultivated for 6 days with addition of either 0,1 or 1µM DAC and DMSO as control. Subsequently, flow cytometric analysis for expression of twelve immune checkpoint molecules (ICM) was performed. OPSCC patients were subdivided by HPV status. **Panel A** shows the fraction of CD4+CD39+ and CD8+CD39+ cells. In CD4+ cells we observed a trend to higher fraction of CD39 expression in HPV negative samples. **Panel B** depicts single ICM expression comparing NC as well as HPV+ and HPV- OPSCC samples. No significant differences were detected after correction for multiple testing. Nevertheless, trends towards higher expression of GITR and OX40 in CD4+CD39+ cells from HPV negative samples were observed. Additionally in HPV- samples, a tendency towards lower expression of TIGIT and CD27 was observed in CD8+CD39- cells and CD4+CD39+ cells, respectively. These findings, in addition to differences compared to NC donors, suggest that the HPV status might influence the ICM repertoire in PBMC derived lymphocytes. Line is drawn at mean with whiskers spanning the 95% confidence interval. Statistical analysis for treatment effects in NC and OPSCC lymphocytes were performed using a Friedemann test, analysis for difference between NC and OPSCC lymphocytes in each treatment group was performed with Kruskall Wallis test. The two-stage linear step-up procedure of Benjamini, Krieger and Yekutieli was used for correction of multiple testing (Q = 0.01). Significant results are marked with asterisks *** = p<0.001 ** = p<0.01.

**Supplementary Figure 4: Effects of DAC on BTLA and TIGIT expression are influenced by donor type.**

Donor lymphocytes were cultivated in vitro for 6 days with Decitabine treatment at 0,1µM and 1µM and DMSO as control. Subsequently cells were assessed flow cytometrically for expression of twelve ICMs. Supplementary Figure 4 shows expression of co-inhibitory ICMs BTLA and TIGIT in CD4+ and CD8+ T-cells. Strikingly we observe predominance of significant BTLA reduction in NC derived samples as well as significant increase of TIGIT expression in OPSCC derived samples. In DMSO treated cells, BTLA was significantly higher expressed in NC samples.

Lines are drawn at mean with whiskers spanning the 95% confidence interval. Statistical analysis for treatment effects in NC and OPSCC lymphocytes were performed using a Friedemann test, analysis for difference between NC and OPSCC lymphocytes in each treatment group was performed with Kruskall Wallis test. The two-stage linear step-up procedure of Benjamini, Krieger and Yekutieli was used for correction of multiple testing (Q = 0.01). Significant results are marked with asterisks *** = p<0.001 ** = p<0.01.

**Supplementary Figure 5: Gating in CFSE Assay**

Frozen donor lymphocytes were thawed, washed and incubated with 1µM of CFSE according to manufacturer’s instructions. Unstimulated, stained as well as unstained, stimulated controls were included. Cells were cultivated and treated as previously described. On seeding day 0 as well as days 3 and 6, cells were harvested, incubated with detection antibodies for discrimination of subpopulations and assessed by flowcytometry. Due to low CFSE concetration clear discrimination between generations was not feasible. Gate 0 harbored all cells on day 0, while non divided cells were observed in gate 1 on days 3 and 6. This was taken into account for calculation of division inices.

**Supplementary Table 1: Antibodies, corresponding isotypes and staining agents used in flow cytometry assays**

**Supplementary Table 2: Cell line information and references**

**Supplementary Table 3: Primer sequences for qRT-PCR of Cancer Testis Antigen RNA**

**Supplementary Table 4: Primer sequences for PyroMark sequencing of CTA promotors**

**Supplementary Table 5: R-Packages and reference**
